# The mediating effect of dispositional mindfulness on the association between UPPS-P impulsivity traits and gaming disorder among Asia-Pacific young adults

**DOI:** 10.1186/s12888-024-05740-0

**Published:** 2024-04-30

**Authors:** Anson Chui Yan Tang, Regina Lai-Tong Lee, Paul Hong Lee, Keiko Tanida, Shun Chan, Simon Ching Lam, Jennifer Nailes, Joy P. Malinit, Jose Ronilo G. Juangco, Qing Wang, Jason Ligot, Lorna Kwai Ping Suen

**Affiliations:** 1 School of Nursing, Tung Wah College, 16/F, Ma Kam Chan Memorial Building, 31 Wylie Road, Hong Kong, China; 2 The Nethersole School of Nursing, Faculty of Medicine, The Chinese University of Hong Kong, Hong Kong, China; 3 School of Nursing and Midwifery, University of Newcastle, NSW, Australia; 4 Southampton Clinical Trials Unit, University of Southampton, Southampton, United Kingdom; 5 College of Nursing Art and Science, University of Hyogo, Hyogo, Japan; 6 Research Institute for Health Sciences, University of the East Ramon Magsaysay Memorial Medical Center, Quezon City, Philippines; 7 Department of Psychiatry, University of the East Ramon Magsaysay Memorial Medical Center, Quezon City, Philippines; 8 College of Medicine, University of the East Ramon Magsaysay Memorial Medical Center, Quezon City, Philippines; 9 School of Nursing, Lanzhou University, Lanzhou, China; 10 College of Public Health, University of The Philippines Manila, Manila, Philippines

**Keywords:** Dispositional mindfulness, Impulsivity, Gaming disorder, Young adult, UPPS-P

## Abstract

**Background:**

Little evidence is available to verify the mediating effect of dispositional mindfulness on the association between gaming disorder and various impulsivity traits. The present study aimed to investigate the mediating effect of dispositional mindfulness on the association between the five UPPS-P impulsivity traits and the risk of gaming disorder among young adults.

**Methods:**

It was an inter-regional cross-sectional study using online survey in Australia, Japan, The Philippines and China. Impulsivity measured by the UPPS-P Impulsive Behavior Scale–Short version; dispositional mindfulness measured by the Mindfulness Attention Awareness Scale; and the risk of gaming disorder measured by the Internet Gaming Disorder Scale were collected in the focal regions. Structural equation modeling was performed by SPSS AMOS version 26 to verify the study hypotheses. Bootstrapped 95% confidence interval was reported. Statistical significance was indicated by the *p*-value below 0.05.

**Results:**

Among the 1,134 returned questionnaires, about 40% of them aged 18–20 years and 21–23 years, respectively. 53.8% were male. 40.7% had been playing digital and video games for over 10 years. The prevalence of gaming disorder was 4.32%. The model fitness indices reflected that the constructed model had an acceptable model fit (χ^2^(118) = 558.994, *p* < 0.001; χ^2^/df = 4.737; CFI = 0.924; TLI = 0.890; GFI = 0.948; RMSEA = 0.058; SRMR = 0.0487). Dispositional mindfulness fully mediated the effect of positive urgency and negative urgency on the risk of gaming disorder. The effect of lack of premeditation on the risk of gaming disorder was partially mediated by dispositional mindfulness. However, dispositional mindfulness did not mediate the effect of sensation seeking on the risk of gaming disorder.

**Conclusions:**

The varied associations between dispositional mindfulness and the five impulsivity traits hints that improving some impulsive traits may increase dispositional mindfulness and so lower the risk of gaming disorder. Despite further studies are needed to verify the present findings, it sheds light on the need to apply interventions on gamers based on their impulsivity profile. Interventions targeting at emotion regulation and self-control such as mindfulness-based interventions seem to be effective to help gamers with dominant features of urgency and lack of premeditation only. Other interventions shall be considered for gamers with high sensation seeking tendency to enhance the effectiveness of gaming disorder prevention.

## Background

Excessive or uncontrollable game-playing, both online and offline, has become a significant public health issue among adolescents and young adults that requires timely identification and management. Internet Gaming Disorder (IGD) has been classified as a kind of behavioural addiction in the Diagnostic and Statistical Manual of Mental Disorders V (DSM-V) since 2013 [1]. To widen the coverage of gaming-related mental disorders, the World Health Organization in 2018 officially defined uncontrollable digital and video gaming as gaming disorder (GD) and filed this disorder in the 11th Revision of the International Classification of Diseases (ICD-11) to reflect its serious health impact and the pressing need for early diagnosis and treatment [2]. A systematic review reported the median prevalence rate of IGD across various countries was 2.0% [3]. In Asia-Pacific region, the prevalence rates were ranged from 2.5 to 34%, which were much higher than the global one [3]. Furthermore, the prevalence of hidden problematic gaming cases in aged 18 or above population could be as high as 38% in some Asian-Pacific countries [4]. It is therefore important to deepen the understanding of the IGD development and identify effective strategies to prevent IGD.

### Impulsivity and gaming disorder

Heightened impulsivity is consistently reported to be positively associated with gaming disorder in systematic review and neuroimaging studies [5, 6]. Impulsivity is defined as a tendency toward rapid, poorly considered and disinhibited decisions and actions, despite negative consequences [7]. The impulsive feature of problematic gamers could be explained by the hypoactivity of the reflective neural system. According to the dual-process model of addiction, addictive behaviours may be a consequence of the imbalanced interaction of the impulsive and reflective neural systems. The impulsive neural system is an implicit cognitive process which triggers automatic responses to behaviour-related stimuli by affect and long-term memory. In contrast, the reflective neural system is an explicit cognitive process targeting at inhibiting response and regulating emotion through conscious deliberation [8]. A recent systematic review examining neurobiological correlates in IGD supported that individuals with IGD had a significant poorer response inhibition and emotion regulation due to the impaired prefrontal cortex functioning and cognitive control [9]. The weakened reflective neural system implies a weaker control on gaming behaviour through a deliberate process. As a consequence, the gamers will engage in gaming uncontrollably due to the progressively stronger desire for gaming as precipitated by the dominant impulsive system over the decision-making process [10].

Most of the existing studies measured impulsivity as a unitary trait [5]. Evidence showed that different dispositions of impulsivity had variant degrees of association with various addictive behaviours and their associated outcomes [7, 11]. The UPPS-P Model of Impulsive Personality has conceptualised impulsivity as a composite of five discrete impulsive personality traits: (1) negative urgency: tendency to act rashly under extreme negative emotions; (2) positive urgency: tendency to act rashly under extreme positive emotions; (3) lack of premeditation: tendency to act without thinking; (4) lack of perseverance: inability to remain focused on a task; and (5) sensation seeking: tendency to seek out novel and thrilling experiences despite its negative consequences [12, 13]. Negative urgency and positive urgency signify the difficulties to control impulsive acts against emotional or behaviour-specific stimuli [12, 14]. Lack of perseverance is related to attentional problems. Sensation seeking and lack of premeditation are related to decision making abilities [14]. A person with greater sensation seeking and lack of premeditation would have difficulties in judging the appropriateness of an action [14]. A systematic review on 32 studies found that the effect sizes between UPPS-P impulsivity traits and alcohol use varied significantly [11]. Rømer Thomsen et al. [7] showed that some UPPS-P impulsivity traits were positively associated with problematic alcohol use, drug use, problematic use of pornography and binge eating in at-risk youth. For example, sensation seeking and lack of perseverance were associated with problematic alcohol use; lack of perseverance was associated with drug use. These traits were not associated with problematic Internet gaming in young adults with no or low risk of Internet gaming disorder [7]. The insignificant influence of UPPS-P impulsivity traits on problematic Internet gaming may be caused by the small sample size in the study (*N* = 109), which was only adequate to detect a modest effect (*r* = 0.35) but not weaker correlations [7]. Costes and Bonnaire [15] conducted a larger study with 3,472 Free-to-Play gamers showed that all the five UPPS-P impulsivity traits except positive urgency were positively associated with GD, with the Odds Ratios of 1.57–2.9. The varied findings among studies for the association between the UPPS-P impulsivity traits and GD are likely due to the issues of sample size and the type of sample examined. As most evidence supports that UPPS-P impulsivity traits have differential strengths of association in different substance and behavioural addictive problems, it is plausible to infer that these traits may also have different strengths of association with GD.

### The mediating role of dispositional mindfulness in the association between impulsivity and the risk of gaming disorder

Dispositional mindfulness is a personality trait that reflects an individual’s tendency to be aware of his thoughts and feelings in the present moment without judgment in daily life [16]. It is modifiable with mindfulness training. Mindfulness is a kind of meditation originating from Buddhist practice to engage an individual in a full, direct and active awareness of experienced phenomena, which is maintained from one moment to the next [17]. The main goal of mindfulness activities is to achieve acceptance of the present moment by paying nonjudgmental attention to events [18]. The emergence of mindfulness-based interventions (MBIs) to manage behavioural addiction including GD is probably due to the core mechanism of mindfulness in fostering self-regulation ability, i.e. response inhibition and emotion regulation [19], which are the major neurological functions found compromised in gamers with heightened impulsivity [9, 20]. Extant literature consistently showed that dispositional mindfulness was positively associated with emotion regulation [21, 22]. It was also found to be a significant mediator for the relationship between difficulties with emotion regulation and substance use disorders [23]. For response inhibition, recent systematic reviews reported that MBIs could enhance inhibitory control with small-to-medium effect sizes in adults [24, 25]. One cross-sectional study showed that dispositional mindfulness was positively related to the accuracy on the inhibitory control task [26].

Some evidence is available to support the associations between impulsivity and dispositional mindfulness [27, 28]. A meta-analysis reported moderate-to-large effect sizes for the negative associations between dispositional mindfulness and negative urgency, positive urgency, lack of premeditation and lack of perseverance [29]. Another study comparing the impulsivity traits between meditators and non-meditators showed that long-term meditators had lower self-reported attentional impulsivity, but higher motor and non-planning impulsivity measured by the Barratt Impulsiveness Scale as compared with non-meditators [30]. It suggested that mindfulness practice may not be effective for all impulsivity traits. It further supported by narrative review and interventional study. Zhang and Zhang [31] conducted a mindfulness-based intervention on adolescents. The structural equation model analysis demonstrated that dispositional mindfulness played a full mediating role in reducing aggression levels of the adolescents. Specifically for GD and other addictive problems, Yao et al. [32] found that decisional impulsivity of the disordered gamers had no significant changes in within-group comparison after receiving the combined virtual reality and mindfulness therapy. A recent narrative review indicated that MBIs were effective for reducing emotion-driven impulsivity such as negative urgency, positive urgency in addictive problems [33].

MBIs have been found to be effective in reducing symptoms of Internet gaming disorder [32, 34], but rarely included dispositional mindfulness as an outcome. A few cross-sectional studies provided support on the association between dispositional mindfulness and GD. Mettler et al. [35] reported that dispositional mindfulness negatively mediated the relationship between problematic gaming and life satisfaction. Chiorri et al. [36] found that problematic gaming behaviour was associated with the lack of acting with awareness facet of dispositional mindfulness measured by Five Facet Mindfulness Questionnaire (FFMQ). Further support could be gained from addictive Internet use. Several related studies have found a negative association of dispositional mindfulness with addictive Internet use [37–39]. For example, Cortazar and Calvete [37] found that the describing and non-judging facets of dispositional mindfulness measured by FFMQ were significantly associated problematic Internet use in adolescents. Song and Park [39] revealed that dispositional mindfulness partially mediated the relationship between stress and Internet addiction.

In sum, existing evidence suggests that different impulsivity traits may have different associations with dispositional mindfulness. The varied associations between impulsivity traits and dispositional mindfulness hint that MBIs may not be effective for all gamers with varied dominant impulsivity traits. It is therefore essential to provide more empirical evidence to reveal the association between dispositional mindfulness, impulsivity traits and gaming disorder. The findings could provide a direction for further investigation on the mechanism of mindfulness training in managing gaming disorder with respect to impulsivity traits.

## Conceptual framework

**Fig. 1:**
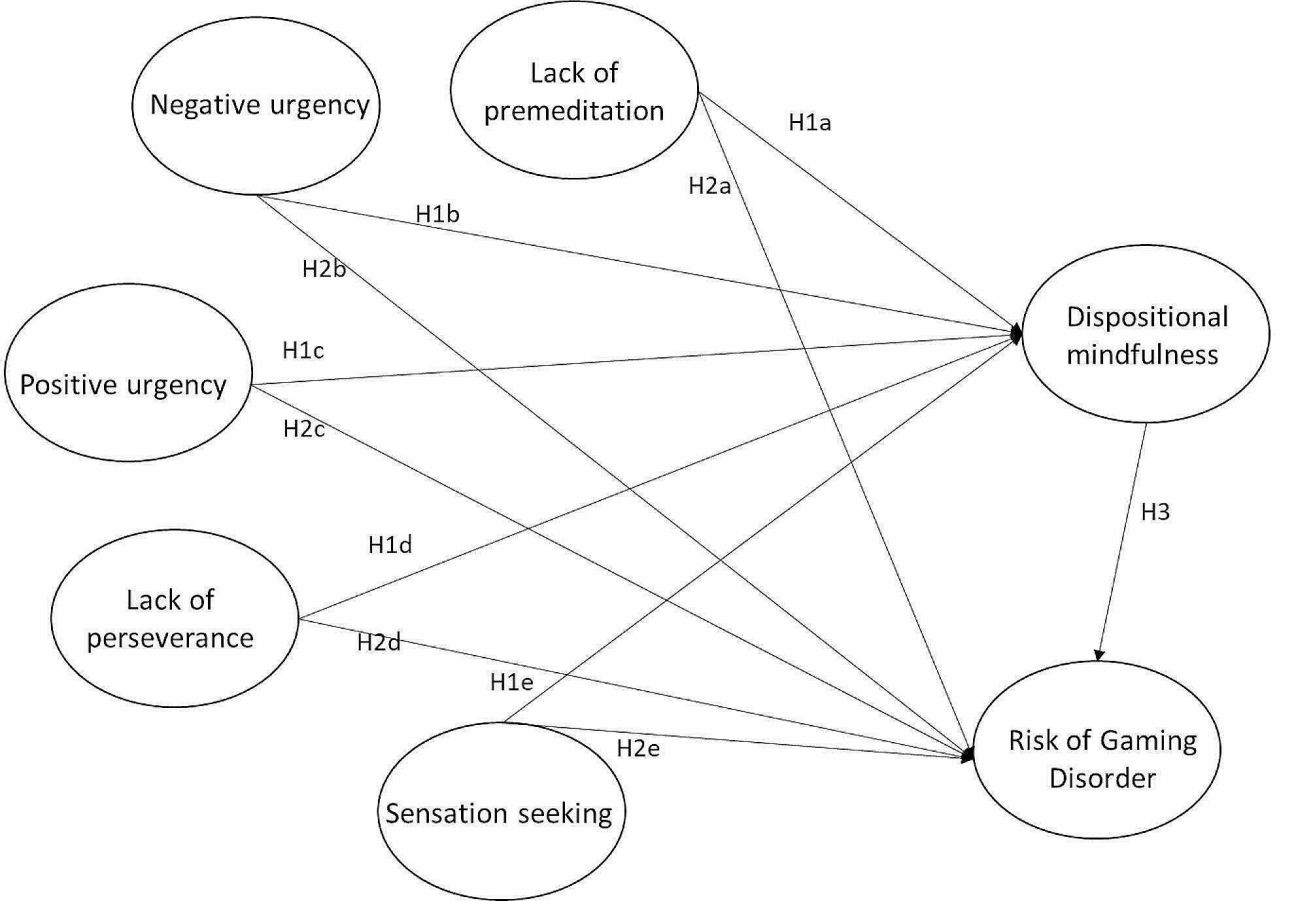
Hypothesized model

The conceptual framework in Fig. 1 depicts the hypothetical associations between the five UPSS-P impulsivity traits, dispositional mindfulness and risk of gaming disorder based on the theoretical mechanism of GD and the existing evidence for the associations among the impulsivity traits and dispositional mindfulness. Four hypotheses were formulated and verified in the present study.

### H1

UPPS-P impulsivity traits, namely lack of premeditation (H1a), negative urgency (H1b), positive urgency (H1c), lack of perseverance (H1d) and sensation seeking (H1e), are positively associated with the risk of GD.

### H2

UPPS-P impulsivity traits, namely lack of premeditation (H2a), negative urgency (H2b), positive urgency (H2c), lack of perseverance (H2d) and sensation seeking (H2e) are negatively associated with dispositional mindfulness.

### H3

Dispositional mindfulness is negatively associated with the risk of GD.

### H4a-e

When mediated by dispositional mindfulness, UPPS-P impulsivity traits, namely lack of premeditation (H4a), negative urgency (H4b), positive urgency (H4c), lack of perseverance (H4d) and sensation seeking (H4e), have indirect effects on the risk of GD.

## Aim of the study

The present study aimed to investigate the mediating effect of dispositional mindfulness on the association between impulsivity traits and GD in young adult gamers using structural equation modelling. This modelling approach helps develop structural relationships among variables having direct and/or indirect effects on GD simultaneously.

## Methods

### Study design and setting

It was an inter-regional, cross-sectional study collecting data using an online survey. Data were collected via an online platform from July 2021 to March 2022 in four countries: Australia, Japan, the Philippines and China. In China, data was collected in two cities: Hong Kong and Lanzhou. The study was approved by the ethics review committee of the respective universities/tertiary institutions.

### Participants and procedure

Convenience and snowball sampling were adopted to recruit young adults aged 18–25 years with experience playing digital or video games over the past 12 months. The exclusion criteria were those with known psychological or mental disorders such as anxiety disorder, and depression or regular use of psychoactive drugs. Sample size estimation was based on one of the common sample size estimation methods in structural equation modelling, i.e. N: q rule, where N is the number of people and q is the number of parameters to be estimated in the model [40]. The recommended ratio of N: q is 20:1 according to Kline (2023) [40]. There were 44 parameters to be estimated in the model, so 880 participants were needed.

The online survey was distributed to the potential participants through universities and tertiary institutions, social media platforms and online gaming platforms in the focal regions. Clicking on the link to the online survey showed information about the study objective, brief procedure and potential benefits on the first page. The survey only began when the participant checked the box for the statement ‘I read through the information above and agree to participate in the study’ as consent to participate. The eligible participants answered questions about demographics and gaming behaviour and rated their risk of GD, dispositional mindfulness and impulsivity using three validated scales. It took 15–20 min to complete the anonymous survey.

### Measures

#### Risk of gaming disorder

The key dependent variable of this study is the risk of GD. It was measured by the nine-item Internet Gaming Disorder Scale first developed by Pontes and Griffiths [41]. The items are derived from the nine IGD diagnostic criteria in the DSM-V [1] because the original scale is used for IGD. The participants rated whether they had these symptoms over the past 12 months on a 5-point Likert scale, where 1 = ’never’ and 5 = ‘very often’ [41]. The total score is the summation of all ratings for the nine items and ranged from 9 to 45. A higher score indicates a higher risk of GD. If the participant has a total score of 32 or above, it is categorised as disordered gaming according to Qin et al. [42]. Examples of items included ‘Do you feel more irritability, anxiety or even sadness when you try to either reduce or stop your gaming activity’, ‘Do you play in order to temporarily escape or relieve a negative mood?’, ‘Have you jeopardised or lost an important relationship, job, or educational or career opportunity because of your gaming activity?’ Chinese and English versions were adapted from Yam et al. [43] and Pontes and Griffiths [41], respectively. Japanese and Filipino versions were translated by the respective research team by forward–backward translation. The internal consistencies of the Japanese and Filipino versions were 0.87 and 0.86, respectively.

#### Impulsivity

Impulsivity was measured by the UPPS-P Impulsive Behavior Scale–Short version [44]. It comprises 20 items measuring five discrete impulsivity traits with four items each. The participants rated their level of agreement with the items on a 4-point Likert scale ranging from 1 (strongly agree) to 4 (strongly disagree). Twelve items are reversely scored. Total impulsivity and trait scores were computed by adding all items and corresponding items, respectively. The total impulsivity score ranges from 20 to 80. Trait scores ranges from 4 to 16. Higher trait scores indicate more inclination to the corresponding impulsivity trait. Examples of items are ‘I generally like to see things through to the end’, ‘My thinking is usually careful and purposeful’ and ‘Unfinished tasks really bother me’. The Chinese, English and Japanese versions were adapted from Xue et al. [45], Cyders et al. [44], and Hasegawa et al. [46], respectively. The Filipino version was translated by the Philippines research team, with a Cronbach’s alpha of 0.68 for the scale and 0.68–0.77 for the five subscales.

#### Dispositional mindfulness

The Mindfulness Attention Awareness Scale, developed by Brown and Ryan [16], was used to measure dispositional mindfulness. It is a 15-item self-report questionnaire measuring people’s tendency to be mindful of moment-to-moment experiences. The participants indicated how frequently they had the experiences described by the items. Each item was measured on a 6-point Likert scale, where 1 = ‘almost always’ and 6 = ‘almost never’. The total score is calculated by the summation of individual item scores divided by 15. The total mindfulness score ranges from 1–6, with a higher score reflecting a higher level of dispositional mindfulness. Examples of items were ‘I could be experiencing some emotion and not be conscious of it until some time later’, ‘I tend to walk quickly to get where I’m going without paying attention to what I experience along the way’ and ‘I find myself doing things without paying attention’. The Chinese, English and Japanese versions were adapted from Chen et al. [47], Brown and Ryan [16], and Fujino et al. [48], respectively. The Filipino version was translated by the Philippines research team, with a Cronbach’s alpha of 0.81.

#### Gaming behaviour

Daily gaming time over the past month and years of gaming were collected and measured as an ordinal scale. Daily gaming time was operationalised as 0–2 h, 3–6 h, 7–10 h and > 10 h. Years of gaming was measured in four categories: 1–4 years, > 4–7 years, > 7–10 years and > 10 years.

#### Demographic variables

Demographic characteristics were collected to learn the baseline characteristics of the participants. Age, gender and education level were collected. Age and education level were measured at an ordinal level, whereas gender was a dichotomous variable.

## Statistical analyses

Frequency and percentage were presented for ordinal and nominal variables including all demographic and gaming behaviour variables. Mean and standard deviation were computed for total GD score, total impulsivity and trait scores, and total mindfulness score. Pearson or Spearman correlation analyses were performed to explore the correlations among all study variables. SPSS version 26 was used to conduct these analyses.

To test the hypotheses, AMOS version 26 was used to conduct structural equation modelling to test the direct and indirect effects of the five UPSS-P impulsivity traits and dispositional mindfulness on the risk of GD. Maximum likelihood estimation was used to examine the model. Bootstrapping procedures were applied to test the direct, indirect and total effects of the seven study variables. To evaluate the model fitness, the following indices were used: goodness-of-fit index (GFI), comparative fit index (CFI) and Tucker-Lewis index (TLI) > 0.9; root mean square error of approximation (RMSEA) and standardised root mean residual (SRMR) < 0.08; and relative chi-square (χ^2^/df) < 5. Statistical significance was defined as a two-tailed *p*-value of < 0.05.

## Results

### Descriptive statistics and correlation

A total of 1,134 questionnaires were received. Table 1 summarises the results for demographic and other study variables. Age groups of 18–20 and 21–23 years each accounted for around 40% of the total participants; 53.8% were male and 46.2% were female. Nearly 70% were undergraduate students. For gaming behaviour, 40.7% had been playing digital and video games for over 10 years. Half of the participants only spent two hours or less playing games every day. Among the participants, 49 (4.32%) were classified as disordered gamers, and 1,085 (95.68%) were classified as normal gamers. The prevalence of GD was 4.32%. Significant differences existed in region, gender and daily gaming time over the past month between normal and disordered gamers. Most of the disordered gamers came from Lanzhou (49%) and the Philippines (40.8%), and nearly 70% were male. They tended to have higher daily gaming times compared with normal gamers; 14.3% of disordered gamers spent seven hours or more gaming every day, whereas only 7.2% and 3.1% of normal gamers spent 7–10 h and > 10 h in gaming a day, respectively.

The total GD score, total mindfulness score and total impulsivity and trait scores were significantly different between groups, except for lack of perseverance. The disordered gamers had significantly greater scores in GD, total impulsivity and its traits than normal gamers. Conversely, the total mindfulness score in disordered gamers was significantly lower than that of normal gamers. This indicated that the disordered gamers tended to exhibit lower mindfulness and greater impulsivity in terms of negative urgency, positive urgency, sensation seeking and lack of premeditation.

**Table 1:** Comparison among the disordered and non-disordered gamers for demographic and independent variables (*N* = 1134)

Study Variables		Total (*N* = 1134)		Normal gamers (*n* = 1085)			Disordered gamers (*n* = 49)		*p* value	
*Demographics*										
Region^a^, n (%)										
Hong Kong		198(17.5)		195(18)			3(6.1)			
Lanzhou		303(26.7)		279(25.7)			24(49)			
Australia		71(6.3)		70(6.5)			1(2)		0.00***	
Japan		198(17.5)		197(18.2)			1(2)			
The Philippines		364(32.1)		344(31.7)			20(40.8)			
Age^^b^, n (%)										
18–20 years		446(39.3)		426(39.3)			20(40.8)			
21–23 years		467(41.2)		449(41.4)			18(36.7)		0.93	
24–25 years		220(19.4)		209(19.3)			11(22.4)			
Gender^a^, n (%)										
Male		610(53.8)		576(53.1)			34(69.4)		0.02*	
Female		524(46.2)		509(46.9)			15(30.6)			
Education level^b^, n (%)										
Primary school or below		11(1)		8(0.7)			3(6.1)		0.14	
Secondary school		171(15.1)		160(14.7)			11(22.4)			
Undergraduate level		790(69.7)		763(70.3)			27(55.1)			
Postgraduate level		162(14.3)		154(14.2)			8(16.3)			
Years of playing digital/video gaming^b^, n(%)										
1–4 years		264(23.3)		252(23.2)			12(24.5)			
> 4–7 years		220(19.4)		212(19.5)			8(16.3)		0.86	
> 7–10 years		188(16.6)		180(16.6)			8(16.3)			
> 10 years		462(40.7)		441(40.6)			21(42.9)			
Daily gaming time over the past month^^b^, n (%)										
0–2 h	571(50.4)				552(50.9)		19(38.8)		< 0.01**	
3–6 h	436(38.4)				420(38.7)		16(32.7)			
7–10 h	85(7.5)				78(7.2)		7(14.3)			
> 10 h	41(3.6)				34(3.1)		7(14.3)			
*Study variables*										
Total GD score^^c^, x̄(SD)		17.01(7.17)		16.18(6.11)			35.37(3.09)		0.00***	
Total mindfulness score^ ^c^, x̄(SD)		3.83(0.89)		3.86(0.87)			3.16(1.09)		0.00***	
Impulsivity score^ ^c^, x̄(SD)										
Total		48.16(8.08)		47.9(8.02)			53.71(7.52)		0.00***	
Negative urgency		10.15(2.67)		10.08(2.63)			11.71(2.82)		0.00***	
Positive urgency		9.68(2.71)		9.61(2.69)			11.14(2.84)		0.00***	
Sensation seeking		10.2(2.94)		10.15(2.94)			11.31(2.87)		< 0.01**	
Lack of perseverance		9.2(2.8)		9.18(2.79)			9.73(2.89)		0.18	
Lack of premeditation		8.9(2.59)		8.86(2.58)			9.82(2.62)		0.02*	

x̄ = mean; SD = standard deviation

GD = Gaming Disorder

^missing data; ^a^Chi-Square test; ^b^Mann-Whitney U test; ^c^Independent t test

*<0.05; **<0.01; ***<0.001

Table 2 shows the correlation analyses between total GD score and age, education level, gaming behaviour, and mindfulness and impulsivity variables. Age was significantly positively correlated with the total GD score, but the strength of correlation was weak (*r* = 0.06, *p* < 0.05). Both daily gaming time and years of gaming were positively correlated with total GD score (*r* = 0.34, 0.2, *p* < 0.001, respectively). The total mindfulness score was negatively correlated with the total GD score (*r* = -0.35, *p* < 0.001). Similarly, total impulsivity and its trait scores were positively correlated with total GD score, except for lack of perseverance. Their correlation coefficients (r) ranged from 0.07 to 0.17, indicating a weak correlation.

**Table 2:** Correlations between total GD score and study variables (*N* = 1134)

	Total GD score	
	*r*	*p *value
Age	0.06	0.03*
Education level	0.05	0.11
Daily gaming time over the past month	0.34	0.00***
Years of gaming	0.2	0.00***
Total mindfulness score	-0.35	0.00***
Total impulsivity score	0.17	0.00***
Negative urgency score	0.14	0.00***
Positive urgency score	0.10	0.00**
Sensation seeking score	0.15	0.00***
Lack of perseverance score	0.04	0.22
Lack of premeditation score	0.07	0.02*

GD = Gaming Disorder

*p < 0.05; **p < 0.01; ***p < 0.001

### Structural model

Out of 1,134 returned questionnaires, 15 contained missing responses to any of the items for dispositional mindfulness, impulsivity and GD. Only 1,119 questionnaires with complete responses were used for computing the structural equation model (SEM). First, confirmatory factor analysis (CFA) was conducted to establish and examine a measurement model for five latent variables (five traits of impulsivity). The results of the CFA indicated that the five impulsivity factors in the hypothesised model fit the data well and gave sufficient measurement validity (χ^2^(94) = 425.32, *p* < 0.001; χ^2^/df = 4.525; CFI = 0.939; TLI = 0.912; GFI = 0.956; RMSEA = 0.056; SRMR = 0.0475).

**Fig. 2:**
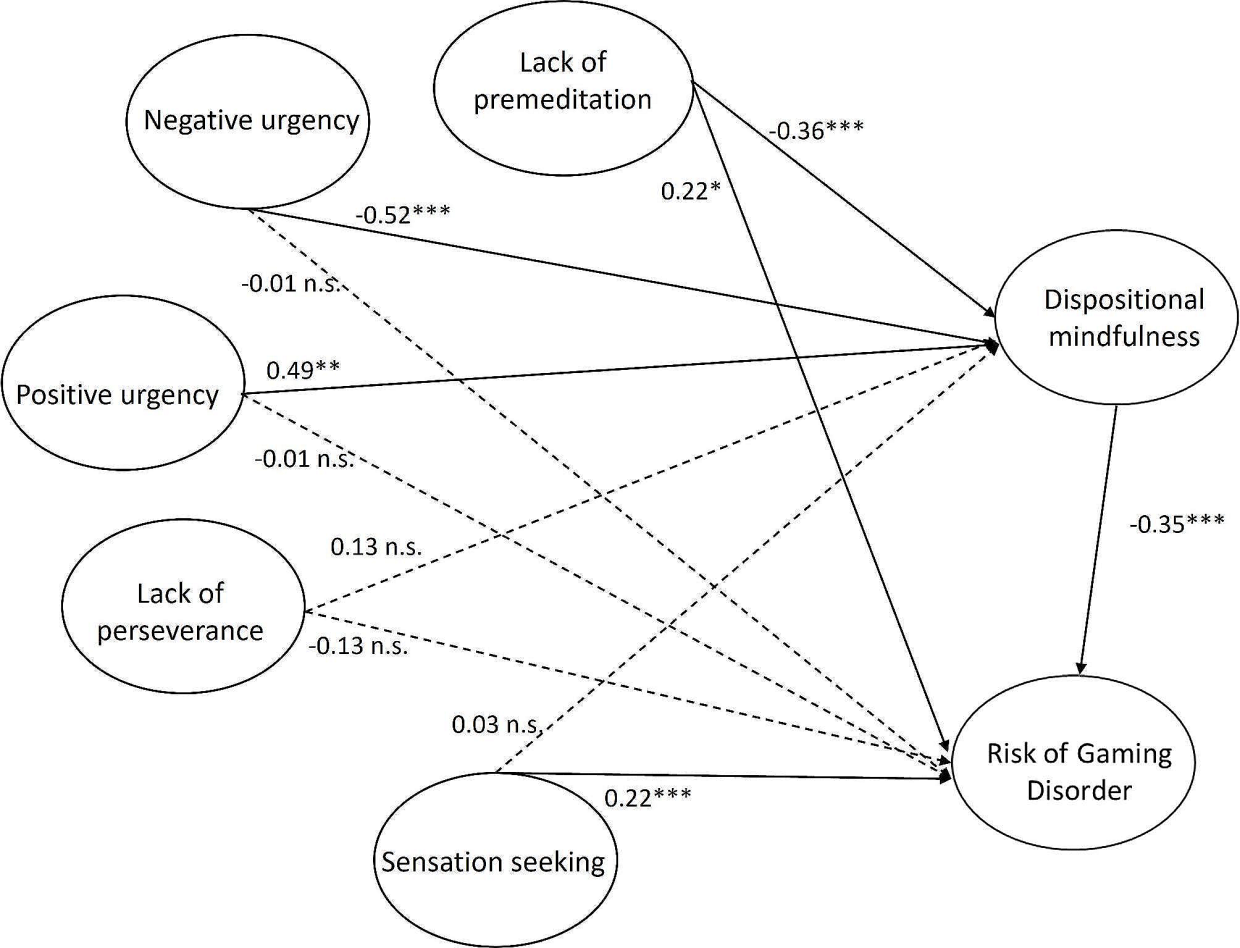
Results of the SEM model forestimating the association among dispositional mindfulness, five impulsivitytraits and risk of Gaming Disorder

A structural model was then established to examine the four research hypotheses. Figure 2 illustrates the SEM results. The model fitness indices reflected that the SEM model had an acceptable model fit (χ^2^(118) = 558.994, *p* < 0.001; χ^2^/df = 4.737; CFI = 0.924; TLI = 0.890; GFI = 0.948; RMSEA = 0.058; SRMR = 0.0487). H1 postulated that the risk of GD was positively associated with lack of premeditation (H1a), negative urgency (H1b), positive urgency (H1c), lack of perseverance (H1d) and sensation seeking (H1e). The results indicated that the total effect of sensation seeking (*β* = 0.21, *p* < 0.01) and lack of premeditation (*β* = 0.34, *p* < 0.001) was positive and significant on the risk of GD. Similar results were found on the direct effect of sensation seeking (*β* = 0.22, *p* < 0.001) and lack of premeditation (*β* = 0.22, *p* < 0.05). H1a and H1e were thus supported. H1d was rejected, though lack of perseverance had a significant total effect on the risk of GD but it was a negative association (*β* = -0.18, *p* < 0.05) and the direct effect was insignificant (*p* = 0.055). H1b and H1c were also rejected because they had no significant total effect (Table 3).

H2 postulated that dispositional mindfulness was negatively associated with lack of premeditation (H2a), negative urgency (H2b), positive urgency (H2c), lack of perseverance (H2d) and sensation seeking (H2e). The results showed that only lack of premeditation, negative urgency and positive urgency showed significant associations with dispositional mindfulness (*β* = -0.36, *p* < 0.001; *β* = -0.52, *p* < 0.001; *β* = 0.49, *p* < 0.01). However, positive urgency had a positive association with the risk of GD. Hence, only H2a and H2b were supported. H3 was supported because dispositional mindfulness was negatively associated with the risk of GD (*β* = -0.35, *p* < 0.001; Fig. 2).

H4 predicted the mediating effect of dispositional mindfulness on the association of the risk of GD and lack of premeditation (H4a), negative urgency (H4b), positive urgency (H4c), lack of perseverance (H4d) and sensation seeking (H4e). According to Table 3, only lack of premeditation had a significant positive indirect effect (*β* = 0.13, *p* < 0.001), direct (*β* = 0.22, *p* < 0.05) and total effects (*β* = 0.34, *p* < 0.001) on the risk of GD. This indicated that dispositional mindfulness partially mediated the relationship between lack of premeditation and the risk of GD. For negative urgency and positive urgency, only the indirect effect was significant (*β* = 0.18, *p* < 0.001; *β* = -0.17, *p* < 0.01). It supported the full mediating effect of dispositional mindfulness on their associations. H4a–c were thus supported. H4d–e were rejected because no significant indirect effects existed for sensation seeking or lack of perseverance (*p* > 0.05; Table 3).

**Table 3:** Bootstrapped results of the SEM model (*N* = 1119)

Independent variable	Indirect effect		Direct effect		Total effect	
	*β*	Bootstrap 95%CI	*β*	Bootstrap 95%CI	*β*	Bootstrap 95%CI
Negative urgency	0.18***	0.08,0.41	-0.01	-0.31,0.35	0.18	-0.1,0.61
Positive urgency	-0.17**	-0.44, -0.05	-0.01	-0.43,0.32	-0.18	-0.65,0.13
Sensation seeking	-0.01	-0.06,0.05	0.22***	0.11,0.33	0.21**	0.09,0.34
Lack of perseverance	-0.04	-0.14,0.02	-0.13	-0.32,0.01	-0.18*	-0.39,-0.03
Lack of premeditation	0.13***	0.05,0.26	0.22*	0.05,0.44	0.34***	0.17,0.59

*p < 0.05; **p < 0.01; ***p < 0.001

## Discussion

This is the first study reporting lack of premeditation, positive urgency and negative urgency as the three impulsivity traits that showed indirect effects on the risk of GD with dispositional mindfulness as a mediator. This is similar to the results of other studies for addiction, such as tobacco use, alcohol use, social media addiction, problematic gambling [49] and problematic mobile phone use [50]. The negative associations between dispositional mindfulness and negative urgency and lack of premeditation echo the findings of a meta-analysis examining the correlations between dispositional mindfulness and impulsivity [29]. It indicated that negative urgency and lack of premeditation had negative correlations with moderate to large mean effect sizes [29].

The impulsive feature of people with greater urgency and lack of premeditation may be related to the weaker prepotent response inhibition and decision-making abilities when facing emotionally arousing conditions because their attention is automatically shifted to the emotionally salient stimuli and behaviour-specific cues according to the Interaction of Person–Affect–Cognition–Execution (I-PACE) model for behavioural addiction [10]. People with weaker prepotent response inhibition are prone to react habitually according to their previous experience and feelings when facing emotional stimuli because these stimuli automatically draw attentional resources [51]. Normally, the selective deployment of attention and emotion to behaviour-specific clues are simultaneously manipulated by the impulsive neural system in the limbic striatal regions, amygdala and orbital-frontal cortex and the reflective neural system in the dorsolateral prefrontal cortex, medial frontal cortex and dorsal anterior cingulate cortex. In at-risk or disordered gamers, the perceptual encoding of emotional stimuli in the amygdala may amplify the early perception of the emotional salience to the behaviour-specific cues due to the subjective reward expectancies of playing games [10, 52]. This may progressively lead to attentional and emotional biases to the behaviour-specific stimuli to fulfil the emotional desire [10, 52]. Consequently, fewer cognitive resources will be available for deliberate control of the behaviour through the reflective neural system.

The lower availability of cognitive resources for the deliberate control of behaviour under emotion-arousing conditions may further precipitate poor decision-making. Urgency-related behaviour was associated with an increased focus on the present emotional needs such as immediate relief of negative emotion, boosting positive emotion regardless of the long-term harmful consequences of the risk-taking behaviour [13]. Some evidence has shown that a low capacity to inhibit prepotent responses was associated with acting without forethought when making decisions in an emotional situation [13]. Findings of neuroimaging studies have revealed that people with greater urgency manifested diminished activities and volumes in brain regions responsible for response inhibition, emotion regulation and emotion-based decision-making, i.e. the orbitofrontal cortex and anterior cingulate dorsomedial prefrontal cortex [53, 54].

The lack of premeditation may be related to the dysfunctional decision-making process [55]. Decision-making is a process in which a choice is made after a conscious and effortful reflection on the consequences of that choice [55]. Damasio [56] explained that decisions may also be determined by an unconscious process driven by somatic markers. Somatic markers refer to physiological reactions associated with anticipatory emotional reactions provoked by a decision that depends on the consequences related to a similar decision in the past [56]. In contrast to voluntary emotion regulation based on deliberate and conscious reflection, people with high negative or positive urgency may tend to regulate emotion in a habitual and unconscious way through the impulsive neural system [52]. So, they tend to rely on the previous feelings and experiences to determine their behaviour in all future similar conditions.

People with greater dispositional mindfulness were found to be less impulsive. This could likely be explained by the emotion-regulatory function of mindfulness [57, 58]. Mindfulness is characterised by the capacity of remaining nonreactive to and accepting distressing thoughts and emotions nonjudgmentally and being aware of automaticity [59]. People with greater dispositional mindfulness may be able to free their minds from being overwhelmed and preoccupied with intense emotions and thoughts because they can reperceive their relationship with thoughts and emotions [60]. Reperceiving emphasises changing one’s relationship with the ephemeral and unreal thoughts and emotions by altering one’s role from doing to being [60]. The reperceiving process could strengthen cognitive flexibility because it increases one’s flexibility of attention and expands one’s metacognitive awareness to reappraise a given event from different perspectives [61]. More cognitive resources, which are usually limited in gamers with high urgency and low premeditation, could then be preserved for reappraising emotion and making decisions through conscious reflection and deliberation [62]. Some studies have reported a positive relationship between cognitive flexibility and dispositional mindfulness [63, 64].

Notably, unlike negative urgency, positive urgency showed a negative indirect effect on the risk of GD, as shown in Table 3. This means that gamers with higher positive urgency would exhibit a lower risk of GD. The negative indirect effect is contributed by the positive association between positive urgency and dispositional mindfulness (Fig. 2). We hesitate on this finding because it may be biased by the sample characteristics. In this present study, most of the participants were normal gamers with no known psychiatric problems. Some studies have reported that positive emotion and a certain degree of impulsivity in healthy individuals could have a robust positive effect on cognitive flexibility and cognitive task performance [65, 66]. In this regard, future studies focusing on disordered gamers are needed to validate the finding.

Sensation seeking was positively associated with the risk of GD, but its effect was not mediated by mindfulness. A recent study exploring associations between UPPS-P impulsivity traits and substance and non-substance addictive behaviours among adolescents and young adults showed that sensation seeking was only associated with alcohol use but not Internet gaming in at-risk youth [7]. This incongruent finding might be due to the different target groups and the small sample size (*N* = 109), which could not detect a small effect size. Other studies have shown that sensation seeking was positively associated with Internet gaming disorder in Chinese, United Kingdom and Arabian adolescents [67–69]. The present finding further confirmed the predictive effect of sensation seeking for increasing the risk of GD in the young adult population. The initial participation in gaming may be provoked by sensation seeking in various game genres to cope with boredom or seeking physio-psychological stimulation to persist positive emotion and counteract negative emotion [67, 70]. Studies have shown that people with a high level of sensation seeking show increased neurobiological responses to intense and novel stimuli [71]. The I-PACE model for behavioural addiction may also explain the findings [10]. At the early stage of behavioural addiction, the desire to play games may be provoked by sensational and emotional gain through the interactive and novel features of various game genres. The generation of positive emotion and experience in gaming may reinforce the gaming behaviour because the experience and emotion generated from game-playing progressively change the subjective reward expectancies associated with gaming and positively reinforce the gamers to continuously engage in this behaviour. The insignificant association between sensation seeking and dispositional mindfulness was congruent with the meta-analysis conducted by Lu and Huffman [29]. Another systematic review showed that there was no clear evidence to support risky decision making could be improved by MBIs [72]. It further affirmed that sensation seeking could not be improved by mindfulness practice. The reason for their insignificant association may be that sensation seeking is processed in the nucleus accumbens and caudate nucleus. Boredom-proneness and fun-seeking individuals were shown to have increased volumes in these two brain regions [73]. To date, no evidence exists to show that mindfulness practice could impose any effect on the activity of these two brain regions.

Lack of perseverance was not associated with GD nor dispositional mindfulness, congruent with previous studies [7]. Lack of perseverance refers to the low tendency to continuously engage in an activity that could be boring or difficult. Some researchers speculated that lack of perseverance may be related to the vulnerability to proactive interference, which is described as the ability to inhibit irrelevant thoughts or memories [55, 74]. People with low perseverance tend to be easily distracted by the environment and irrelevant thoughts, which could slow down their performance in the recognition paradigm tasks [75]. According to the I-PACE model, game-playing is initially driven by fun-seeking and other emotional factors that may, in turn, reduce cognitive control due to progressive attentional and emotional biases to emotional salience stimuli [10]. Therefore, whether gamers manifest a low ability to persist tasks may not be a direct determining factor for GD, although a significant difference existed in the lack of perseveration between normal and disordered gamer groups in the present study. Furthermore, the majority of the participants were normal gamers, which may undermine the effect of lack of perseverance on GD, given that the *p*-value of the direct effect for the association between lack of perseverance and risk of GD was 0.055.

### Limitations and future directions

Although the current study contributes to a better understanding of impulsivity traits, dispositional mindfulness and GD, our results should be interpreted with caution because this study has several limitations. First, our model explains the relationship between mindfulness, impulsivity traits and GD based on cross-sectional data. The causal relationships among them could not be inferred. Future studies should adopt a longitudinal design to establish causality. Second, the validity of the study findings may be undermined by the data collection method, i.e. all measures were collected through self-reports. Response and recall biases cannot be excluded. Further studies may include objective data such as the Stroop test or multiple questionnaires for the same construct to triangulate the self-report data. Third, this study focused on testing the roles of dispositional mindfulness and impulsivity on GD. Future studies should explore the potential moderation effect of some demographic factors such as gender and daily gaming time on the significant associations revealed in the established model. Additionally, different facets of mindfulness may have different associations with each impulsivity trait, as found in previous studies such as Peters et al. [27]. Future studies should investigate the associations of different facets of mindfulness on GD and impulsivity to deepen our understanding on the effect of mindfulness on the relationship between GD and impulsivity. Finally, the model established was based on a dataset with a 95% response from normal gamers. The imbalanced samples between normal and disordered gamers may undermine the effects of some impulsivity traits on GD. Future studies should obtain a more balanced sample size for both normal and disordered gamers to verify the findings.

### Implications for practice

Despite these limitations, this study offers important insights into an under-investigated area. It is the first study exploring the association of GD with individual impulsivity traits rather than a unitary approach with a large sample size in five Asia-Pacific regions. It deepens the understanding of the connection between GD and impulsivity, which is a well-known risk factor strongly associated with problematic gaming. The principal findings of the present study suggest that dispositional mindfulness could mediate the effect of lack of premeditation, negative and positive urgency on the risk of GD but not lack of perseverance and sensation seeking. It adds to the existing understanding that gamers with variant dominance in any impulsivity traits could interact differently with dispositional mindfulness. Interventions targeting at emotion regulation and self-control, commonly MBIs, may only be effective for gamers with high urgency and lack of premeditation. It implicates that healthcare professionals should apply interventions on gamers based on their impulsivity profile. Other non-mindfulness-based interventions shall be explored for gamers with high sensation seeking tendency to enhance the effectiveness of gaming disorder prevention. From a research perspective, the findings might help researchers to further refine the theoretical model for GD and differentiate it from other behavioural addictive disorders given different behavioural addictive problems showed different associations with the impulsivity traits. It may also facilitate the classification of gamers into subtypes, which is a key direction for the field [76].

## Conclusion

The present study showed that UPPS-P impulsivity traits had variant degrees of association with the risk of GD. Dispositional mindfulness could partially mediate the effect of lack of premeditation and fully mediate the effect of negative and positive urgency on the risk of GD. The findings deepen the present understanding of the effect of impulsivity and dispositional mindfulness on GD. This could facilitate healthcare professionals and researchers to explore effective interventions other than mindfulness-driven to prevent and manage GD with respect to the dominant impulsive feature of individual gamers.

## Data Availability

The datasets used and/or analysed during the current study are available from the corresponding author on reasonable request.
